# Risk stratifiers for arrhythmic and non-arrhythmic mortality after acute myocardial infarction

**DOI:** 10.1038/s41598-018-28327-8

**Published:** 2018-07-02

**Authors:** Alfonso M. Gañán-Calvo, Katerina Hnatkova, Álvaro Romero-Calvo, Juan Fajardo-López, Marek Malik

**Affiliations:** 1 Universidad de Sevilla, Depto. de Ingeniería Aeroespacial y Mecánica de Fluidos, Sevilla, E-41092 Spain; 2 Imperial College, National Heart and Lung Institute, London, SW3 6LY United Kingdom; 3 Lynzos SL, Engineering Department, Sevilla, E-41018 Spain; 4 Clínica HLA Santa Isabel, Intensive Care Unit, Sevilla, E-41018 Spain

## Abstract

The effective discrimination between patients at risk of Arrhythmic Mortality (AM) and Non-Arrhythmic Mortality (NAM) constitutes one of the important unmet clinical needs. Successful risk assessment based on Electrocardiography (ECG) records is greatly improved by the combination of different indices reflecting not only the pathological substrate but also the autonomic regulation of cardiac electrophysiology. This study assesses the cardiac risk stratification capacity of two new Heart Rate Variability (HRV) parameters, Breath Concurrence 6 (BC6) -sinusoidal RR variability of 6 heart beats per breath cycle- and Primary Ectopia (PE) -presence of early ventricular contractions of any etiology- together with the Deceleration Capacity (DC). While BC6 characterizes the response to physiological and pathophysiological stimuli, PE qualifies autonomic cardiac electrophysiology. The analysis of the European Myocardial Infarct Amiodarone Trial (EMIAT) database indicates that BC6 is related with the risk of Arrhythmic Mortality (AM) and PE with the risk of Non-Arrhythmic Mortality. BC6 is the only single parameter that significantly discriminates between AM and NAM. While the combination of BC6 and DC contributes to the identification of AM risk, PE together with DC improves the prediction of NAM in patients with severe ischemic heart disease.

## Introduction

Identification of patients at increased risk of death is a multifactorial process. It depends not only on factors that cannot be modified, such as age, sex, comorbidity, and the injury severity, but also on factors that might be altered by disease progression, treatment delay, and therapeutic effects on the target organ and on the whole organism.

The problem of mortality stratification among cardiac patients also involves the distinction between the risk of Arrhythmic Mortality (AM) and Non-Arrhythmic Mortality (NAM)^1^. Patients at increased risk of AM might benefit from Implantable Cardioverter Defibrillators (ICD) although not all AM are preventable by ICD interventions. On the contrary, ICD therapy is less likely effective in patients at increased risk of NAM. However, the present guidelines for the selection of candidates for ICD implantation are based on low Left Ventricular Ejection Fraction (LVEF) and do not make much distinction between AM and NAM risk^2^.

This is now well recognized and effective: identification of patients at risk of AM rather than of NAM constitutes one of the important unmet clinical needs^3^. Multiple studies aimed at addressing this need and involved a variety of non-invasive risk profiling techniques. A consensus emerges from these studies that successful risk assessment of cardiac patients needs to involve combination of different indices reflecting not only the pathological substrate but also autonomic regulation of cardiac electrophysiology^4^.

To contribute to the development of multifactorial risk assessment in cardiac patients, we present a combination of a newly developed Heart Rate Variability (HRV) assessment mode^5^ with Deceleration Capacity (DC)^6^, which is one of the most powerful risk predictors based on HRV principles. We aim at demonstrating that even within the HRV framework, combination of different indices may advance the field of cardiac risk stratification meaningfully.

## Methods

### Population and long-term electrocardiographic recordings

To evaluate the differences in AM and NAM risk prediction based on HRV assessment, data of cardiac patients with objective distinctions between different mortality modes are needed. For this purpose, we used the database of the previously conducted European Myocardial Infarct Amiodarone Trial (EMIAT)^7^ which enrolled post-Myocardial Infarct (MI) patients with LVEF not exceeding 40% and randomized these to placebo and prophylactic amiodarone treatment.

The EMIAT trial was appropriately ethically approved and all participants gave informed consent which included further research use of their data. The trial patients were followed up for 2 years (median follow-up of 21 months) and all death cases were classified as non-cardiac (e.g. due to malignancies), cardiac non-arrhythmic (e.g. due to slow progression of heart failure) and cardiac arrhythmic. The classification was performed by a dedicated committee of the trial and the classifications were available for the purposes of the present study. All methods were performed according with the approved guidelines and regulations described by Julian *et al*.^7^.

The combination of the new HRV method with DC was tested in the placebo part of the EMIAT trial. Per protocol, 24-hour Holter recordings were obtained in all trial patients. In addition to these Holter recordings, LVEF measurements in the patients were also available for the purposes of the present investigation.

In all Holter recordings, detailed sequences of RR intervals including the distinction between sinus-nodal and ectopic beats were available as previously described. The data of DC measurement in the recordings (as briefly described further) were available from previous analyses of the parameter.

The novel HRV indices were evaluated by the University of Seville team without any access to the follow-up data. The follow-up data were kept by the Imperial College London team who were involved in the evaluation of the indices calculated by the Sevilla team but were kept blinded in terms of the nature of the novel HRV indices.

### Assessment of deceleration capacity

DC values were obtained by means of the previously described technology^6^. Briefly, the DC index was calculated using the following steps:

1. **Definition of the anchors**: RR intervals longer than the preceding interval are identified as anchors.
2. **Definition of segments**: Segments are defined as RR interval vectors around the anchors. All segments have the same size, chosen according to the lowest frequency to be visualized.
3. **Phase rectification**: All segments are aligned with the anchors as reference point.
4. **Signal averaging**: A *X*(*i*) signal is obtained by averaging the segments. *X*(0) is the average of the RR intervals at the anchors, *X*(1) is the average of the RR intervals immediately following the anchors, *X*(−1) is the average immediately preceding the anchors, etc.
5. **Quantification of DC**: The DC coefficient is obtained as

1 $$DC=\frac{1}{4}[X\mathrm{(0)}+X\mathrm{(1)}-X(-\mathrm{1)}-X(-\mathrm{2)}]\mathrm{.}$$

### Assessment of Breath Concurrence and Primary Ectopia

The novel HRV assessment was based on previously described principles^5^, summarized as follows. A Holter Record RR report is here treated as a set of *M* consecutive values corresponding to the measured RR intervals, that can be expressed as ${\{{X}_{i}\}}_{i\mathrm{=1,...,}M}$. To perform a study of the existence of universal HRV sequences and their appearance, the record can be represented as a locally normalized set of consecutive vectors

2 $${\delta }_{i}={\{\frac{{X}_{i+j}}{{\langle X\rangle }_{i,N}}-1\}}_{j\mathrm{=0,...,}N-2},$$

where *N* is the length of the sequence to be analyzed, and ${\langle X\rangle }_{i,N}={N}^{-1}{\sum }_{j\mathrm{=0}}^{N-1}{X}_{i+j}$ is the local average^5^. In this work, while the average is taken for *N* subsequent beats, only *N* − 1 components of ${\delta }_{i}$ are considered, since the *N*th-component of ${\delta }_{i}$ would equal the sum of the first (*N* − 1) ones according to the local normalization made. This normalization supposes an important step to analyze the presence of characteristic structures among individuals, independently of their average beat rate or instantaneous activity. Among the infinite diversity of possible structures, a limited number has been observed in real records^5^. Ones of the most common structures found are lines that pass through the origin of coordinates. Thus, it is possible to define the simplest universal sequence as a line in an (N−1)-dimensional space of the form

3 $$A=\{{a}_{1},{a}_{2},\,\mathrm{...,}\,{a}_{N-1}\}t,$$

parametrized by a variable *t* representing the severity of heart rate variability.

Two sequences of this form were defined on a previous publication, where the first one is given by

4 $$B{1}_{5}=\{-\mathrm{1,}\,\mathrm{1,}\,\mathrm{0,}\,0\}t,$$

and represents an ectopic beat which produces an early beat (−1)*t* followed by a beat delayed by an increment in time twice as wide as the advance in previous beat (+1)*t*, and two subsequent regular beats (0). Note that *t* times the corresponding component of the vector is the normalized time difference found between the corresponding beat and the average of the interval (the base of normalization). The majority of ectopic beats siting around *B*1_5_ had ventricular origin.

The second sequence is defined by

5 $${{\rm{Breath}}}_{N}={\{-\sin (\frac{2\pi (j+\mathrm{1)}}{N})\}}_{j\mathrm{=0,...,}N-2}t,$$

and reflects the modulation of breath on heart rate variability. This sequence assumes that a complete breath cycle takes approximately *N* heart beats. A previous study has shown that the presence of this sequence is approximately maximized for *N* = 6 in healthy individuals^5^, a value which is assumed for this study. Hence, the corresponding sequence is

6 $${{\rm{Breath}}}_{6}=\{-\frac{\sqrt{3}}{2},-\frac{\sqrt{3}}{2},0,\frac{\sqrt{3}}{2},\frac{\sqrt{3}}{2}\}t\mathrm{.}$$

The projection of the *δ* vectors on those sequences makes it possible to define the generalized deviation angles

7 $${\theta }_{i}={|{\cos }^{-1}(\frac{A\cdot {\delta }_{i}}{\Vert A\Vert \cdot \Vert {\delta }_{i}\Vert })|}_{i\mathrm{=1,...,}M},$$

where “·” denotes the scalar product of two vectors, ||  || their module, and |  | the absolute value. The quantity

8 $$\frac{A\cdot {\delta }_{i}}{\Vert A\Vert \cdot \Vert {\delta }_{i}\Vert }$$

can also be understood as the Pearson correlation coefficient between the real and the predetermined heart beat sequence.

The presence of events (% of sequences) where the deviation angle ${\theta }_{i}$ is below a predetermined level ${\theta }_{max}$ is a coefficient which measures the occurrence of the corresponding sequence, i.e.:

9 $$P({\rm{ \% }})=100\cdot \frac{1}{M}\sum _{i=1}^{M}(1\,{\rm{i}}{\rm{f}}\,{\theta }_{i}\le {\theta }_{max};\,0\,{\rm{o}}{\rm{t}}{\rm{h}}{\rm{e}}{\rm{r}}{\rm{w}}{\rm{i}}{\rm{s}}{\rm{e}}).$$

We performed a blind-blind analysis where the two previous parameters were calculated for each individual. A tolerance of ${\theta }_{max\mathrm{,1}}=0.05$ was selected for the calculation of the presence of $B{1}_{5}$, while ${\theta }_{max\mathrm{,2}}=0.2$ was set for ${{\rm{Breath}}}_{6}$. The choice of these tolerances for each sequence was based on a selection not influenced by subsequent results, and therefore subject to further optimization. In this work, that choice was based on a statistical analysis both intra-sample and among samples:

1. Each individual shows an increasing presence of points (vectors) around the corresponding sequence as the volume defined by the angle *θ* increases, until a first plateau at *θ*_*tp*_ is reached. Above that turning point *θ*_*tp*_, the number of vectors that fall inside the volume defined by *θ* > *θ*_*tp*_, increases at a significantly smaller pace. That turning point *θ*_*tp*_ is rather sharp in about 90% of individuals with a presence of the corresponding sequence above 10% (“positives”).
2. Since *θ*_*tp*_ varies among individuals, we selected an approximate overall value *θ*_*max*_ below 0.25 and above the average *θ*_*tp*_ of the 90% of positives with smaller *θ*_*tp*_, corresponding to that around which the number of positives presented a minimal variation.

**Figure 1:**
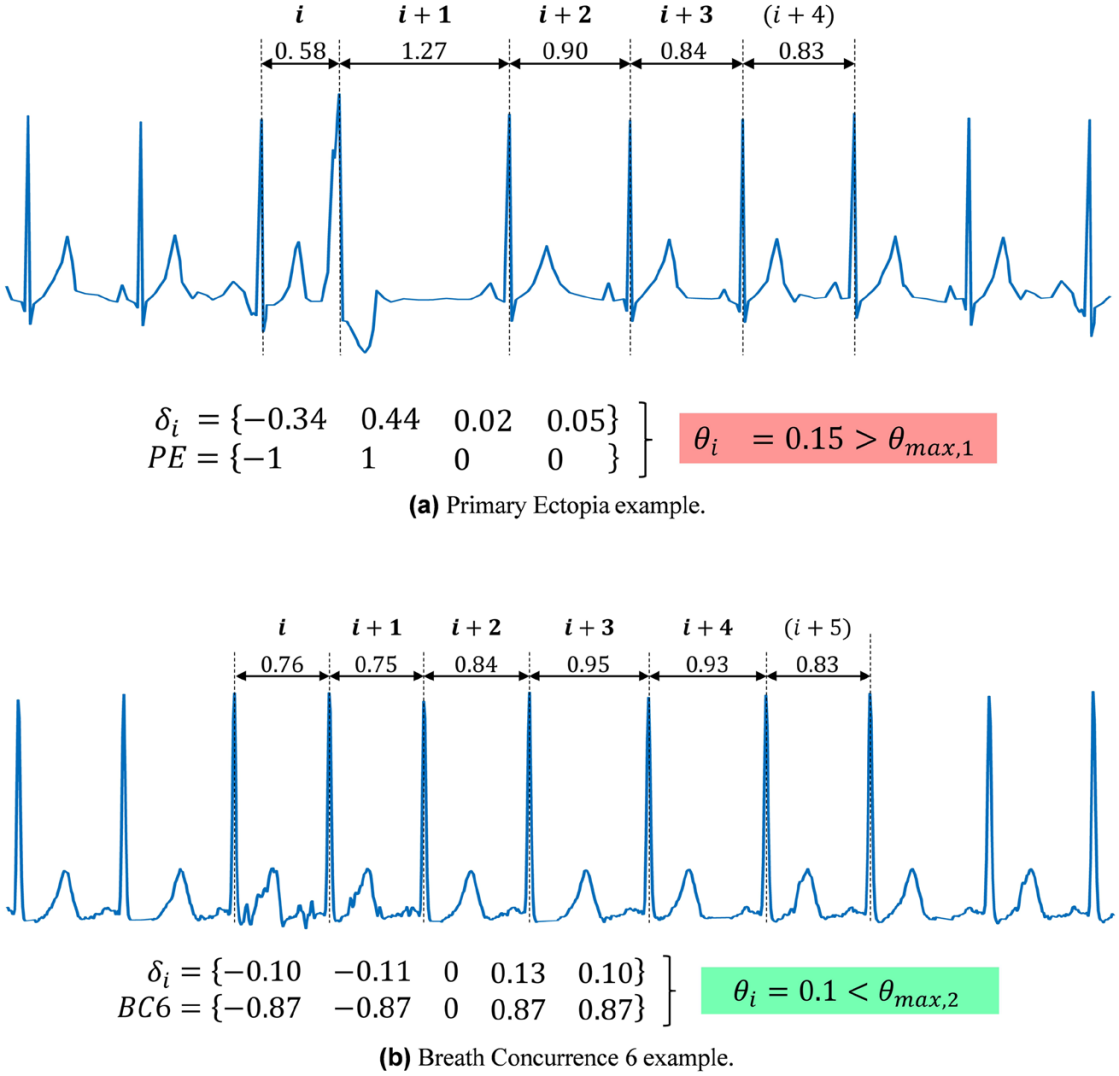
Synthetic Electrocardiography (ECG) curves showing (**a**) a PE example outside tolerance and (**b**) a BC6 example inside tolerance. The components of vector *δ*_*i*_ are calculated according to equation (2), while *θ*_*i*_ is given by equation (7).

From now on, the presences of *B*1_5_ and ${{\rm{Breath}}}_{6}$ with selected tolerances ${\theta }_{max\mathrm{,1}}$ and ${\theta }_{max\mathrm{,2}}$ will be called **Primary Ectopia** (PE) and **Breath Concurrence 6** (BC6), respectively. A conceptual scheme of the calculation process is given in Fig. 1.

These coefficients were calculated by the research group in Sevilla, Spain, based solely on the sequences of normal-to-normal RR intervals in the long-term EMIAT trial Holter records. We emphasize that the research group in Sevilla was (and still is) fully blinded in terms of the identity, clinical characteristics, and follow-up outcome of individual EMIAT patients. On the contrary, while performing the statistical analyses, the research group in London, England, was fully blinded in terms of the principle and calculations of the detailed indexes.

Given the nature of our blind-blind analysis here reported, the two tolerances ${\theta }_{max\mathrm{,1}}$ and ${\theta }_{max\mathrm{,2}}$ were not optimized to provide the highest significance in the results per se. A subsequent study analyzing the sensitivity of the significance of the results to these tolerances shall be performed in the future.

### Statistics and data presentation

Where appropriate, data are presented as mean ± standard deviation. Comparisons of LVEF, DC, PE, and BC6 were made between survivors and patients who, during the follow-up, died of All Cause Mortality (ACM), NAM, and AM. Two-sample two-tail t-test assuming unequal variances were used for this purpose.

Firstly, the risk prediction by LVEF, DC, PE, and BC6 were compared univariately including the dependency of mortality modes on the distribution of these indices. For this purpose, the complete study population was sorted according to each of the indices. The mortality (including the proportion between AM and NAM) was evaluated in different parts of the population ranging from 10% of the highest risk patients (according to the given index) up to 100% of the complete population. These dependences were compared graphically.

Subsequently, mortality prediction by LVEF, DC, PE and BC6 was tested by multivariate Cox regression model. Two different variants of the model were used, one using a combination of LVEF, DC, and PE; the other using a combination of LVEF, DC, and BC6. These Cox models were performed for ACM, NAM, and AM.

Finally, to concentrate on the assessment based on the Holter analyses, a dichotomy of DC, PE, and BC6 was found selecting (for each of these indices) one third of the patients with the highest risk of ACM. Probability of ACM, NAM and AM was compared between the dichotomized parts of the population as well as for a combination of the dichotomized parts by two of the indices using Kaplan-Meier survival curves. Cases of two Kaplan-Meier curves were compared by log-rank tests; cases of more curves were compared by chi-square tests.

Statistical evaluations were made using SPSS Statistics package release 24.0.0.1. P-values below 0.05 were considered statistically significant.

## Results

### Population and survival data

Holter recordings were available in 634 of EMIAT patients randomized to the placebo group. During the 2-year follow-up of the trial, 87 of these patients died. Of these cases, 12 were non-cardiac (e.g. due to malignancies), 32 were cardiac non-arrhythmic (e.g. due to slow progression of heart failure), and 43 were cardiac arrhythmic mortalities. The application of the previously defined coefficients to the placebo patients of the EMIAT database results in a 634 × 2 matrix of values which are subsequently analyzed.

### Univariate risk prediction

Table 1 shows the comparison of the evaluated indices in patients who survived during the follow-up period and those who died, including ACM, NAM and AM cases. The table shows that similar to LVEF and DC, the PE and BC6 parameters were statistically significantly different between survivors and non-survivors, increased values of PE were predominantly found in patients at risk of (counterintuitively) NAM while decreased values of BC6 were predominantly found in patients at risk of AM. Of all the investigated parameters, BC6 index was also the only one that statistically significantly differed between patients who subsequently succumbed to NAM and AM.

**Table 1:** Comparison of LVEF, DC, PE, and BC6 between patients who survived (S) during the follow-up and those who died (ACM, AM, NAM).

	LVEF [%]	DC	PE	BC6
Survivors	30.55 ± 7.28	4.69 ± 3.55	0.79 ± 2.27	1.04 ± 0.63
All-Cause Mortality	26.52 ± 7.91	2.25 ± 4.41	1.81 ± 3.01	0.87 ± 0.62
p-value S vs ACM	<**0.0001**	<**0.0001**	**0.0031**	**0.0224**
Non-Arrhythmic Mortality	24.84 ± 8.61	2.68 ± 3.25	2.21 ± 3.71	1.06 ± 0.61
p-value S vs NAM	**0.0008**	**0.0019**	**0.0395**	0.8371
Arrhythmic Mortality	26.88 ± 7.53	1.53 ± 5.43	1.55 ± 2.71	0.76 ± 0.62
p-value S vs AM	**0.0033**	**0.0005**	0.0782	**0.0071**
p-value NAM vs AM	0.1873	0.2132	0.4566	**0.0296**

As shown in Table 1, compared to survivors, patients at increased mortality risk had smaller LVEF, smaller DC, larger PE, and smaller BC6.

**Figure 2:**
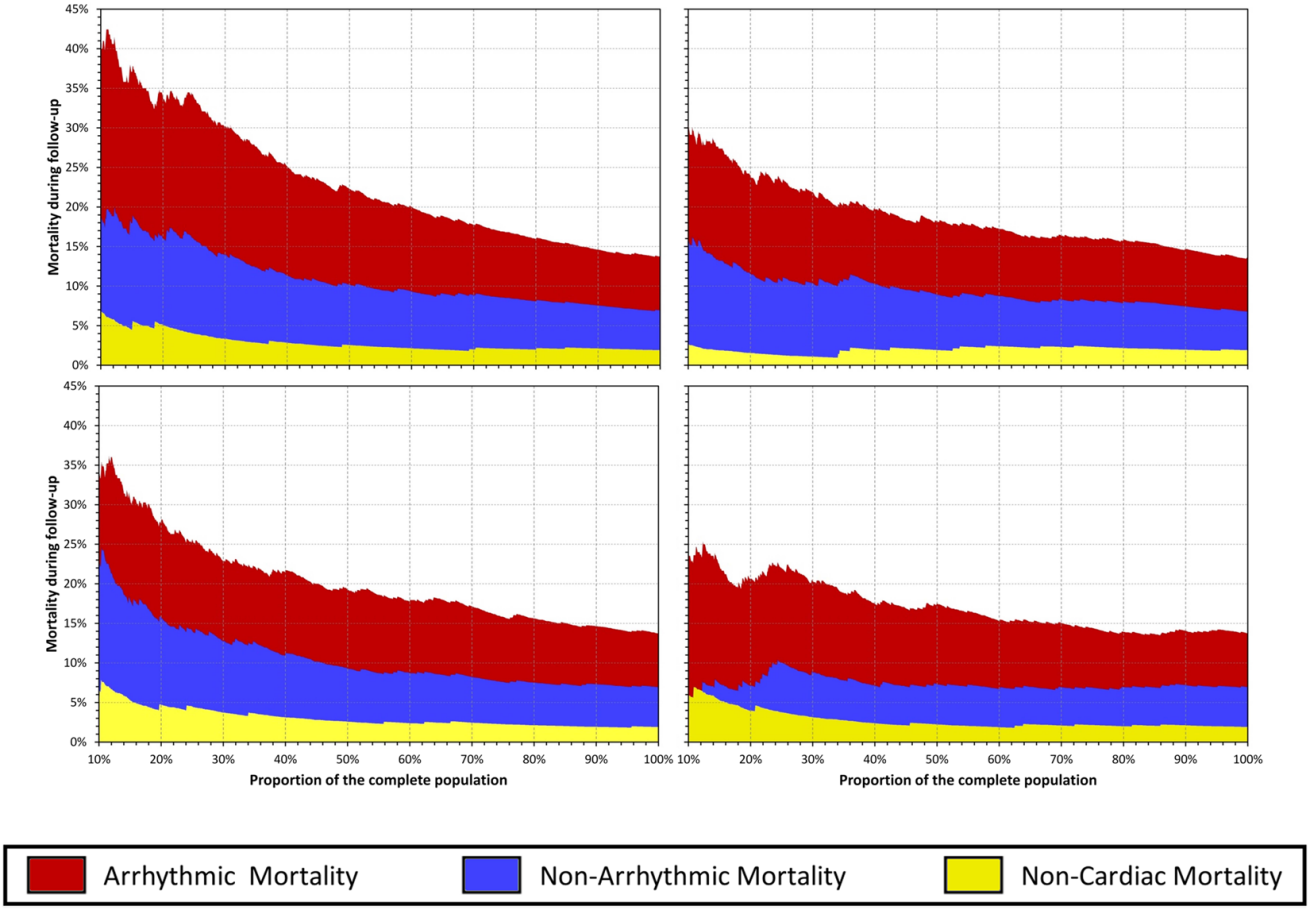
Progression of mortality risk when stratifying high risk patients based on the coefficients DC, LVEF, PE and BC6.

**Figure 3:**
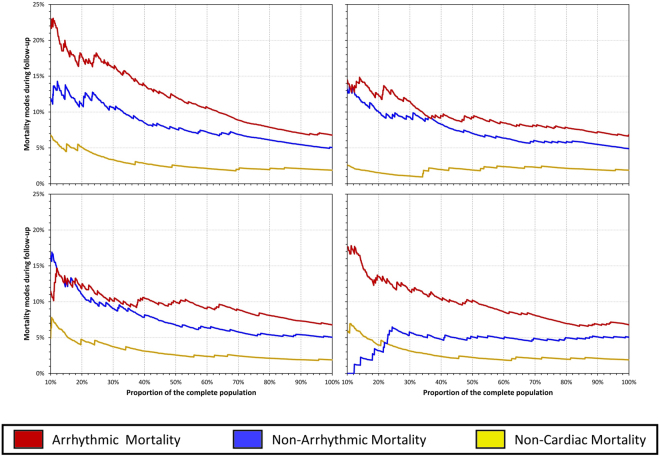
Separate progression of mortality risk when stratifying high risk patients based on the population subgroups with lower DC, LVEF, PE and BC6 coefficients.

Figures 2 and 3 consequently show the mortality levels in different subgroups of high risk patients – i.e. patients with smallest LVEF, DC or BC6 and largest pe, respectively. While Fig. 2 shows the different mortality modes cumulatively, Fig. 3 shows the mortality strata separately. The comparisons of the risk stratifiers shown in Figs 2 and 3 are in agreement with the statistical evaluations presented in Table 1. In particular, Fig. 3 shows that the increase of mortality risk among patients with the smallest values of BC6 is almost exclusively caused by an increase of AM.

**Figure 4:**
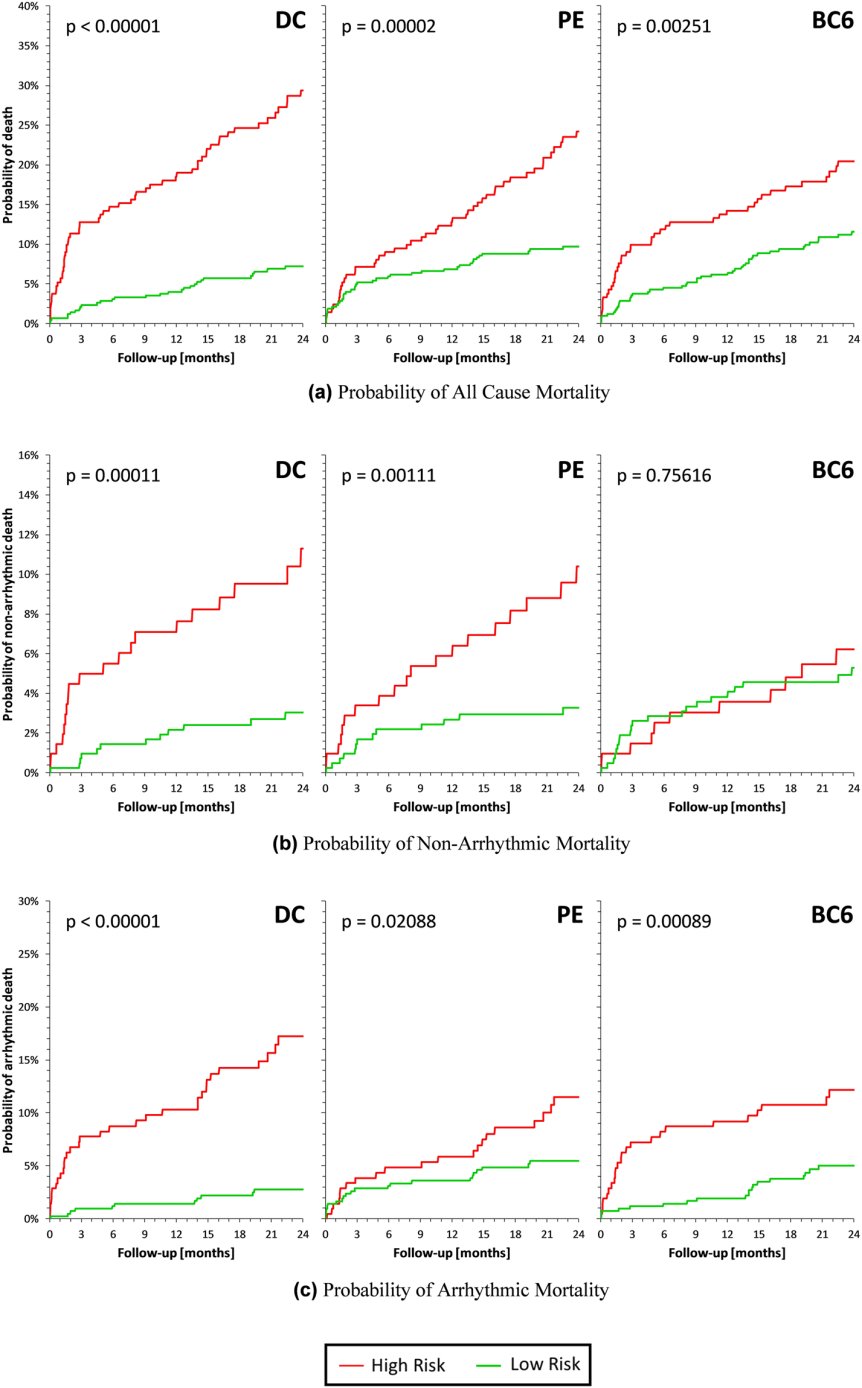
Stratification of DC, PE and BC6 coefficients at one third of the data for (**a**) ACM, (**b**) NAM and (**c**) AM. Label indicates the color codes for each group considered. High Risk group is the 33% highest mortality one, while Low Risk group is the 66% lowest mortality one.

The univariate Kaplan-Meier curves of the probabilities of death stratified according to DC, PE, and BC6 are shown in Fig. 4. In each of the cases, one third of the populations at high risk of ACM is compared to the two thirds of the population at lower risk of ACM.

The graphs in Fig. 4 suggest that while PE is comparable to DC in terms of the prediction of NAM, BC6 is comparable to DC in terms of the prediction of AM.

### Multivariate risk prediction

Table 2 shows the significances of the two Cox proportional hazard models based on continuous risk stratifies as well as using the 33% high-risk dichotomies of the stratifiers. The trends of the comparisons of PE and BC6 for the prediction of NAM and AM correspond to those observed in the univariate analysis.

**Table 2:** Statistical significances of Cox proportional hazard models. For each risk stratifier and for each follow-up outcome, the top line of the results shows the results of Cox proportional hazard models applied to continuous numerical data while the bottom line (in italics) shows the results of the models applied to the parameters dichotomized at one third of the population (not optimized for either PE or BC6 yet). P-values are shown together with Hazard Ratios (HR) and with their 95% Confidence Intervals (CI). The bold results indicate statistical significance at p < 0.05.

**Model 1**	p-value	LVEF		p-value	DC		p-value	PE	
		HR	HR 95% CI		HR	HR 95% CI		HR	HR 95% CI
ACM	<**0.001**	**0.950**	**0.926–0.975**	<**0.001**	**0.921**	**0.882–0.962**	0.834	1.007	0.946–1.071
	***0.032***	***0.626***	***0.408–0.959***	<***0.001***	***0.272***	***0.168–0.441***	*0.075*	*0.664*	*0.432–1.042*
NAM	<**0.001**	**0.924**	**0.886–0.963**	0.523	0.969	0.881–1.066	0.195	1.072	0.965–1.190
	***0.019***	***0.424***	***0.207–0.869***	***0.020***	***0.397***	***0.182–0.866***	*0.061*	*0.484*	*0.227–1.035*
AM	**0.030**	**0.959**	**0.924–0.996**	<**0.001**	**0.891**	**0.848–0.936**	0.408	0.961	0.874–1.056
	*0.263*	*0.707*	*0.386–1.297*	<***0.001***	***0.161***	***0.078–0.335***	*0.882*	*0.953*	*0.508–1.790*
**Model 2**	**p-value**	**LVEF**		**p-value**	**DC**		**p-value**	**BC6**	
		**HR**	**HR 95% CI**		**HR**	**HR 95% CI**		**HR**	**HR 95% CI**
ACM	<**0.001**	**0.950**	**0.926–0.975**	<**0.0001**	**0.928**	**0.894–0.964**	0.210	0.784	0.536–1.147
	***0.024***	***0.611***	***0.398–0.938***	<***0.001***	***0.251***	***0.159–0.397***	***0.022***	***0.608***	***0.398–0.930***
NAM	<**0.001**	**0.927**	**0.889–0.966**	**0.026**	**0.925**	**0.863–0.991**	0.408	1.266	0.724–2.212
	***0.018***	***0.419***	***0.204–0.860***	***0.001***	***0.304***	***0.146–0.634***	*0.993*	*0.997*	*0.477–2.081*
AM	**0.021**	**0.957**	**0.922–0.993**	**0.001**	**0.921**	**0.878–0.967**	0.080	0.590	0.327–1.065
	*0.234*	*0.691*	*0.376–1.270*	<***0.001***	***0.177***	***0.088–0.357***	***0.010***	***0.451***	***0.246–0.826***

**Figure 5:**
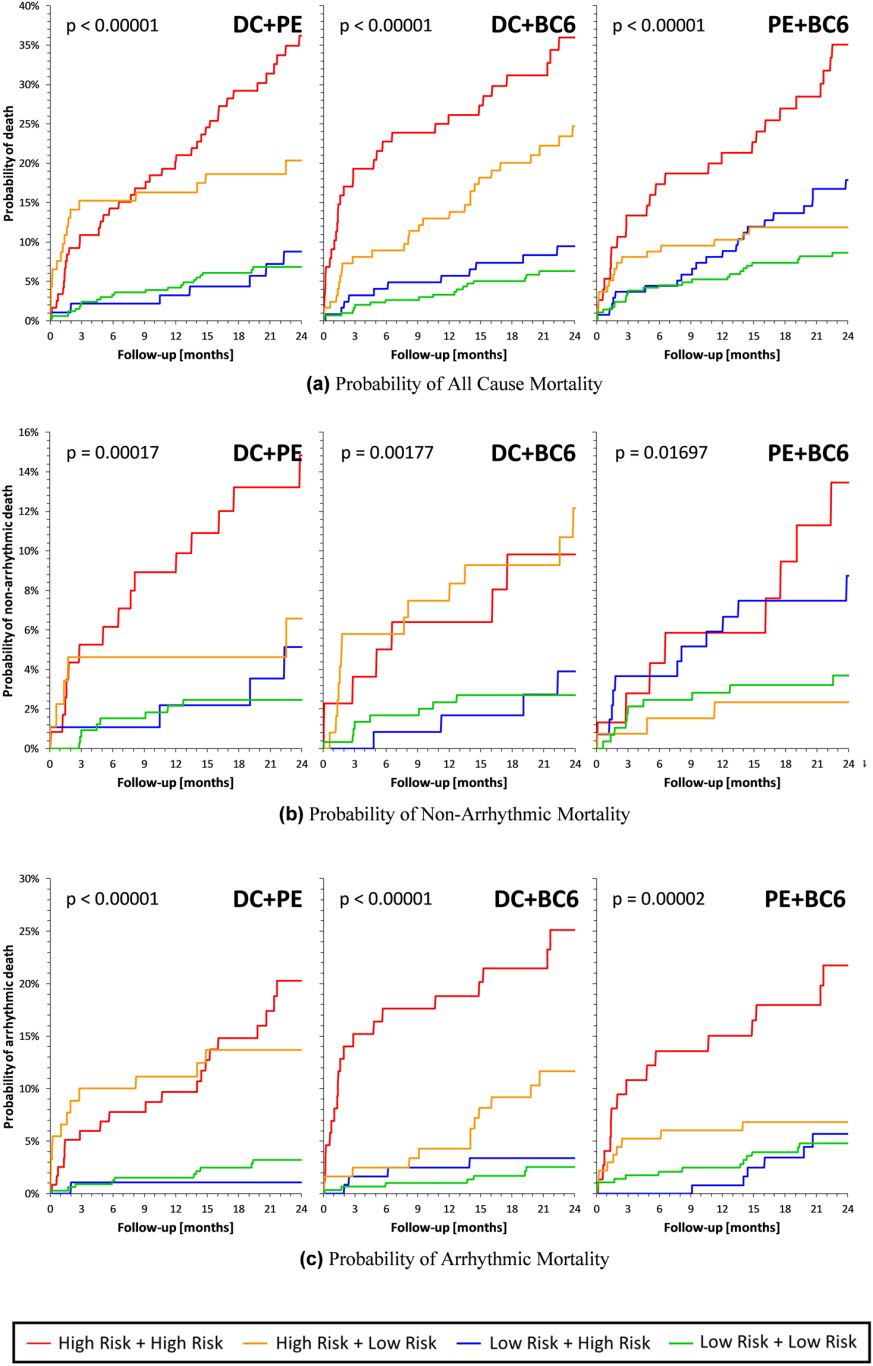
Survival curves for the combinations (DC + PE), (DC + BC6) and (PE + BC6) when using the 33% dichotomies for (**a**) ACM, (**b**) NAM and (**c**) AM. Label indicates the color codes for each two-groups of the combination considered. High Risk group is the 33% highest mortality one, while Low Risk group is the 66% lowest mortality one.

Kaplan-Meier curves of probabilities of death, NAM, and AM for multivariate combinations between DC, PE, and BC6 are shown in Fig. 5.

The Figure shows that combining high risk signified by both BC6 and DC leads to a powerful prediction of AM. On the contrary, combination of high risk signified by both DC and PE leads to powerful prediction of NAM albeit the percentage level of predicted NAM is lower than that of the AM. Still, comparison of Figs. 4 and 5 shows that addition of BC6 and PE to DC leads to meaningful increase in the predicted level of AM (in case of BC6 addition) and NAM (in case of PE addition) in comparison to DC alone. Besides, the addition of BC6 and PE also leads to strong prediction of AM.

## Discussion

It is not surprising that absolute separation of AM and NAM was not achieved in our analyses. AM is caused by ventricular fibrillation, ventricular tachycardia or asystole/pulseless electrical activity, whereas heart failure is the main cause of NAM. Complex factors involved in the risk stratification give rise to a continuous risk spectrum. This spectrum is modulated by environmental and genetic factors and by biorhythms^8,9^. The determination of an isolated risk is further complicated by competing risk: patients at risk of AM are also subject to a risk of NAM and non-cardiovascular mortality. Different death modalities share risk factors such as functional status, delays in intraventricular conduction and concomitant atrial fibrillation^10–12^.

Individual risk factors, such as ventricular performance^13^ and abnormal autonomic regulation^14,15^ that appear immediately after an MI, change over time. In a 10-year follow-up of 120 patients with ischemic heart disease and LVEF < 50%, no difference was found in the cumulative incidence of AM among patients with left ventricular dysfunction (LVEF < 30%) and without it (>30%)^16^. Patients with LVEF > 30% have a good initial prognosis, but after 2 years of follow-up, the risk of AM increases progressively until reaching the same level of risk as patients with depressed left ventricular function.

New HRV parameter BC6 characterizes sinus-nodal response to physiological and pathophysiological stimuli. It has previously been found to progressively decrease in the presence of cardiac abnormalities^5^. PE is related to the presence of early ventricular contractions. The association of these indices with different mortality modes appears physiologically plausible.

Although the application of the new HRV parameters to the placebo patients of the EMIAT trial does not seem to significantly improve the individual prediction of mortality, a strong relationship between BC6 and AM has been shown. BC6 was the only parameter able to significantly discriminate between AM and NAM.

The new HRV parameter PE, although being related with the ectopic activity, has not shown a prognostic capacity with AM, but with NAM, as previously reported. In heart failure the highest PE and the lowest BC6 were found^5^.

Our results indicate that the combination of different novel HRV parameters with the well-established DC risk indicator might contribute to the separation of AM and NAM in patients with severe ischemic heart disease. Since these factors are derived from Holter recordings, adding the novel HRV parameters to DC does not imply additional clinical burden in the process of selecting high-risk patients. Although what the new parameters add to DC in this limited study may appear modest in the pure multivariate statistical sense, they may contribute meaningfully to the practical selection of specific high-risk groups.

### Limitations

There are several important limitations of this study that need to be considered. Whilst we used the EMIAT trial data because of the objective and independent classification of mortality modes in a relatively high-risk population (patients with LVEF above 40% were not enrolled), the data are historical and were obtained during the era of pharmacological thrombolytic treatment before the introduction of routine acute angioplasty procedures (almost two thirds of the patients reported in this sub-study received acute thrombolysis). It is possible if not likely that with acute angioplasty interventions, the levels of mortality risk are presently somewhat lowered. Nevertheless, it is also likely that the distinction between AM and NAM is presently similar to what it was during the EMIAT trial^17^. Both DC and the novel HRV parameters utilize RR interval sequences. Electrocardiographic recordings obviously contain further information such as on ventricular repolarization and on QRS abnormalities which have previously been also used for risk prediction^18,19^ and which could thus also be used in multivariate settings. The setup of the novel HRV parameters can also be further optimized. The tolerances ${\theta }_{max\mathrm{,1}}$ and ${\theta }_{max\mathrm{,2}}$ have been selected on the basis of those approximate minimum values below 0.25 where the first plateaus of the corresponding presence *P*(%) and the number of individuals with that presence above 10% are both reached. This selection has been made independently of the results obtained, but it can be subject of subsequent optimization in future works for risk prediction applications.

## Conclusion

All retrospective analyses can naturally be only hypothesis generating. Nevertheless, the recently developed novel HRV methods appear to contribute to the risk prediction in severe ischemic heart disease. In particular, the novel technology provided, among others, two coefficients related to the breath influence on HRV and the presence of ectopic beats. This study has shown that their combination with a well-known risk predictor of DC can improve significantly the AM and NAM risk separation on patients who survive the acute phase of MI.
